# A Hydrotalcite-Based PET Composites with Enhanced Properties for Liquid Milk Packaging Applications

**DOI:** 10.3390/ma16051857

**Published:** 2023-02-24

**Authors:** Xiangnan Feng, Xiaomeng Hu, Jie Yu, Min Zhao, Fan Yang, Xinrui Wang, Caili Zhang, Yunxuan Weng, Jingbin Han

**Affiliations:** 1 College of Chemistry and Materials Engineering, China National Light Industry, Beijing Technology and Business University, Beijing 100048, China; 2 State Key Laboratory of Chemical Resource Engineering, Beijing University of Chemical Technology, Beijing 100029, China

**Keywords:** PET, layered double hydroxide, gas barrier, UV resistance, antimicrobial, packaging

## Abstract

In the present work, the two-phase mixture (HTLc) of hydrotalcite and its oxide were used to improve the barrier properties, UV resistance and antimicrobial activity of Poly(ethylene terephthalate) (PET) for their application in liquid milk packaging. Firstly, CaZnAl-CO_3_-LDHs with a two-dimensional layered structure were synthesized by hydrothermal method. CaZnAl-CO_3_-LDHs precursors were characterized by XRD, TEM, ICP and dynamic light scattering. A series of PET/HTLc composite films were then prepared, characterized by XRD, FTIR and SEM, and a possible mechanism of the composite films with hydrotalcite was proposed. Barrier properties to water vapor and oxygen have been studied in PET nanocomposites, as well as their antibacterial efficacy by the colony technique and their mechanical properties after exposure to UV irradiation for 24 h. By the presence of 1.5 wt% HTLc in the PET composite film, the oxygen transmission rate (OTR) was reduced by 95.27%, the water vapor transmission rate was reduced by 72.58% and the inhibition against Staphylococcus aureus and Escherichia coli was 83.19% and 52.75%. Moreover, a simulation of the migration process in dairy products was used to prove the relative safety. This research first proposes a safe technique for fabricating hydrotalcite-based polymer composites with a high gas barrier, UV resistance and effective antibacterial activity.

## 1. Introduction

The basic role of dairy packaging, as well as any other food product, is to provide a physical barrier to prevent different damages to the product (mechanical, physical, microbial contamination, etc.) and to maintain optimal product quality [1,2,3]. At present, dairy packaging materials are generally divided into two categories: composite material of paper, plastic or aluminum and multilayer co-extruded film [4]. Poly(ethylene terephthalate) (PET) is a high polymer produced by the dehydration of ethyl terephthalate synthesis reaction and is one of the most diffused thermoplastic polymers available on the market [5,6]. It has some very useful properties for food packaging, such as transparency, chemical resistance, good recyclability and mechanical properties. PET bottles have been a popular product in the milk packaging sector in Europe and the US in recent years. However, in recent years, consumers have demanded healthy and safe lifestyles and the changes in people’s daily life due to COVID-19; consequently, the trend in the food industry is to maximize freshness and avoid synthetic preservatives. Therefore, the modification of PET is necessary. Many efforts have been made to improve the barrier, efficient antibacterial activity and food preservation characteristics properties of PET. Active packaging with efficient antibacterial activity and food preservation characteristics has attracted scientists [7,8]. Li et al. [9] inserted the antimicrobial agent menthoxytriazine into the backbone of PET, named PEMT. Antibacterial adhesion test and antifungal landing test exhibited that PEMT had better resistance to Escherichia coli (Gram-negative), Staphylococcus aureus (Gram-positive) and Aspergillus niger (fungi) contamination compared to PET. The main commercial approaches to improve the gas barrier properties of PET rely on the postprocessing application of impermeable coatings or blending PET with other high-barrier polymers [10]. Wang [11] and his team prepared whey protein isolate/chitosan/microcrystalline cellulose composite PET bottles for rosebud beverage packaging. The results showed that multilayer PET bottles can significantly improve gas barrier performance and extend the shelf-life of rosebud beverages. Aili Wang et al. [12] added a photoprotectant (TiO_2_) to conventional PET bottles to reduce the rate of LED light-induced oxidation in milk. The combination of TiO_2_ and oxygen barrier properties (PET) successfully protected the freshness of milk from photo-induced oxidation even at high LED light intensities. However, almost none of the above studies have improved the overall performance of PET to meet the needs of PET bottles in liquid milk packaging. Therefore, there is an urgent need to develop a new PET material with a strong gas barrier, light barrier and UV resistance, and efficient antibacterial activity that can preserve liquid milk for a long time without deterioration.

The use of functional additives might improve the crystallinity, thermal stability, antibacterial activity and barrier property of PET at the same time. Among the inorganic fillers that can modify the polymers’ properties and functionalities, layered double hydroxides (LDH) are very attractive and versatile [13]. Divalent and trivalent metal hydroxides are covalently bonded to each other to form the main lamellae and are rich in positive charge, while interlayer anions are arranged in an orderly manner to balance the charge of the main lamellae with electrostatic force, and the two are alternately stacked to form two-dimensional layered materials [14,15].

Therefore, it can be anticipated that the incorporation of LDH into a PET matrix will give rise to one kind of novel packaging for liquid milk with the following advantages. First, the presence of the LDH host matrix can serve as a UV blocker and may reduce light-induced oxidation of extended shelf life milk. Second, LDH host layers impose an isolation effect within the PET substrate and provide a barrier structure, which could extremely prolong the path length for the diffusing gas. Finally, the LDH with Zn element may improve the antimicrobial activity of PET packaging. This system could potentially increase the stability and specificity of preservation and reduce the number of chemicals needed in foods.

The aim of the present work was to improve the performance of PET films for liquid milk packaging applications. For this purpose, a series of PET/HTLc composite films were prepared by a hot pressing method, and the effect of HTLc content on their structures and properties was investigated. The main focus was placed on the barrier properties, antibacterial activity and food preservation safety properties of the composite films. This work provides a new strategy for the design and preparation of dairy packaging materials.

## 2. Materials and Methods

### 2.1. Materials

All the chemicals used were of analytical grade or the highest purity commercially available. A superfine powder PET, with an intrinsic viscosity of 0.83–0.87 dL/g and a melting point of 247 °C, was obtained from the Dow. Ca(NO_3_)_2_·4H_2_O, Zn(NO_3_)_2_·6H_2_O, Al(NO_3_)_3_·9H_2_O, NaOH and Na_2_CO_3_ (A.R.) were purchased from Sinopharm Chemical Reagent Co., Ltd., (Beijing, China)

### 2.2. Synthesis of CaZnAl-CO_3_-LDHs

A salt solution containing Ca(NO_3_)_2_·4H_2_O, Zn(NO_3_)_2_·6H_2_O, Al(NO_3_)_3_·9H_2_O and another alkaline solution containing NaOH (2 mol/L) and Na_2_CO_3_ (0.5 mol/L) were simultaneously dripped into a beaker containing a small amount of deionized water at a rate of one drop per second. The total metal ion concentration of the added salt solution was maintained at 1.0 mol/L, and the molar ratios of Ca/Zn/Al were 3.6:0.4:2. At the same time, the obtained mixed solution was vigorously stirred at 65 °C for 30 min, and the reaction pH was controlled at 10 value. Then, the resultant mixture was transferred into a Teflon-lined autoclave and heated at 120 °C for 24 h. After cooling to room temperature, the gained product was filtered, washed three times with deionized water and ethyl alcohol and dried at 60 °C for 24 h, followed by grinding to fine powder [16].

### 2.3. Preparation of PET/HTLc Nanocomposites

The nanocomposite film was prepared using a melt hot press procedure without solvents and monomers, which has environmental and cost advantages. Firstly, CaZnAl-LDHs and PET were dispersed uniformly under the action of a suspended stirrer IKA RW20. The powder mixture was dried under vacuum at 140 °C for 24 h to remove moisture and then pressed under vacuum at 20 tons for 300 s between two nonstick metal sheets using a ZG-20T press. The melt temperatures during nanocomposite film preparations were equal to 280 °C. When preparing the nanocomposite film, the CaZnAl-LDHs contents were varied between 0.5, 1, 1.5, 2, 2.5 and 3 wt%. When the addition amount was 3 wt%, the mechanical properties of the composite film decreased significantly, and it was almost impossible to form a film. Therefore, the gradients of 0.5%, 1%, 1.5%, 2% and 2.5% were selected for the later characterization and testing, and the samples were designated as PET/HTLc-0.5, PET/HTLc-1, PET/HTLc-1.5, PET/HTLc-2 and PET/HTLc-2.5, respectively.

### 2.4. Characterization

Powder X-ray diffraction (XRD) measurements were measured using an X-ray powder diffractometer (Bruker D2, Karlsruhe, Germany) under the conditions of 30 kV, 10 mA, Cu/Kɑ radiation (λ = 1.5406 Å), with a scanning rate of 5° min^−1^) and 2*θ* covered the range of 5–80°.

Scanning electron microscopy (SEM) image was acquired on JSM 6700F scanning electron microscopy with an operating voltage of 10 kV. High-resolution transmission electron microscopy (HRTEM) studies were performed at the JEOL JEM-ARM200F at an acceleration voltage of 200 kV.

FT-IR spectra were obtained on a Thermo Scientific Nicolet IS 10 instrument in the attenuated total reflectance (ATR) mode.

The particle size and zeta potential were measured by a ZetasizerNano ZS90 (Malvern Instruments, Malvern, UK) with a scattering angle of 90° at 25 °C.

The thickness of the film was measured with a CHY-C2A pachymeter (Labthink Instruments Co., Ltd. Jinan, China).

Thermo-gravimetric (TG) analysis was performed on a TGA Q5000IR instrument (TA Instruments, New Castle, DE, USA) in the temperature range of 23–800 °C, and the heating rate of 10 min^−1^ in N_2_ purging flow was 40 mL/min.The differential scanning calorimetry (DSC) measurements were performed using a Q20 differential scanning calorimeter (TA Instruments).

The chemical state of the sample was examined by X-ray photoelectron spectroscopy (XPS), performed with ESCALAB 250Xi (Thermo Fisher, Waltham, MA, USA).

### 2.5. Gas Barrier

Based on the differential pressure method, the gas O_2_ and water vapor transmission rates were measured using a VAC-V2 and W3/060 gas transmission rate testing system (Labthink Instruments Co., Ltd., Jinan, China). Oxygen transfer rate (OTR) was tested at 23 °C and 0% RH, and water vapor transfer rate (WVTR) was tested at 38.1 °C, 90% RH. All OTR and WVTR values were averaged from at least three separate films.

### 2.6. UV Aging Resistance

Ultraviolet-visible (UV-vis) absorption spectra of the samples were collected for the spectral range 200–800 nm using a SolidSpec-3700i/3700i DUV−vis spectrometer with BaSO_4_ as a reference.

The UV aging experiments were conducted in a UV light box with a wavelength of 365 nm, and the optical power density was controlled at 9.26 w/m^2^. A series of composite films were subjected to UV irradiation for 24 h to record the tensile strength before and after aging, and three parallel groups were set up.

The maximum tensile strength (Rm) and maximum deformation of a series of PET/HTLc films before and after UV light aging were tested using an electromechanical universal testing machine (CMT 6104, MTS Systems Co., Ltd., Shanghai, China) equipped with a tensile load cell of 100 N. The cross-head speed was set at 50 mm min^−1^. Each sample was tested at least three times [17].

### 2.7. Antimicrobial Testing

The antibacterial properties of the HTLc were first measured using the colony method. Gram-positive bacteria Staphylococcus aureus (*S. aureus*, ATCC 6538) and Gram-negative bacteria Escherichia coli (*E. coli*, ATCC 8739) microorganisms were measured by counting colony forming units (CFUs) using the tilted plate technique. The strain was inoculated in nutritional broth (NB) at 37 °C for 12 h. The Yang strain was washed 3 times with phosphate-buffered saline (PBS), diluted with PBS, and had an optical density of 1.0 at 600 nm (OD600), approximately 1 × 109 CFU/mL. Then 500 mL of the mixture consisting of 100mL of the above bacterial suspension, different amounts of the nanocomposite and PBS were added to a 1.5 mL centrifuge tube, which was cultured in an incubator at 37 °C for 30 min. The suspension was serially diluted 2.5 × 10^5^ times with PBS. An amount of 100 mL of the diluted suspension was transferred to a Luria–Bertani (LB) medium nutrient agar plate and incubated in an incubator at 37 °C for 24 h. The inhibition capacity was directly monitored by calculating the amount of CFU on the LB nutrient agar plate. The antibacterial rate of the nanocomposite was calculated using Equation (1) as follows:

Antibacterial rate (%) = (N_0_ − N)/N_0_ × 100 (1)

where N and N_0_ are the number of CFU of the bacteria with and without nanocomposites, respectively.

A membrane PET/HTLc-1.5 with the best combined gas barrier and UV resistance was selected for the colony method of bacterial inhibition experiments. Briefly, clean samples of 2 cm × 2 cm were heat sterilized at 121 °C for 20 min, washed with sterilized PBS and dried. The samples were placed in 2 mL of bacterial suspension (1 × 10^9^ CFU/mL) and incubated at 37 °C for 24 h, after which the bacterial suspension was serially diluted with PBS as in the previous method, inoculated into LB plates, incubated, the number of colonies counted and the inhibition rate calculated [18]. All antibacterial tests were performed in three parallel experiments.

An amount of 100 µL of bacterial suspension and 8 cm^2^ of PET/HTLc-1.5 film were added to 3 mL of liquid medium. The mixture of liquid medium and bacterial suspension without the composite membrane was used as the control. The culture was incubated continuously for 15 h at 37 °C and 110 r/min. After that, the supernatant was collected by centrifugation at 4000 r/min for 5 min. The contents of K^+^ and Mg^2+^ in the two supernatants were determined by an inductively coupled plasma emission spectrometer (ICP). The protein content of the two supernatants was determined by BCA protein concentration assay [19].

### 2.8. Safe Migration Experiments

Two sheets of 1 dm^2^ PET/HTLc-1.5 films were completely immersed in 100 mL of milk and yogurt, respectively, and left airtight at 37 °C for 10 days. After 10 days, 1 mL of yogurt and milk were taken separately for nitration, after which the contents of Ca, Zn, Al and their isotopes in the solutions were measured by ICP to determine the migration of LDHs [20].

## 3. Results and Discussion

### 3.1. Characterization of CaZnAl-CO_3_-LDHs and PET/HTLc

**Figure 1:**
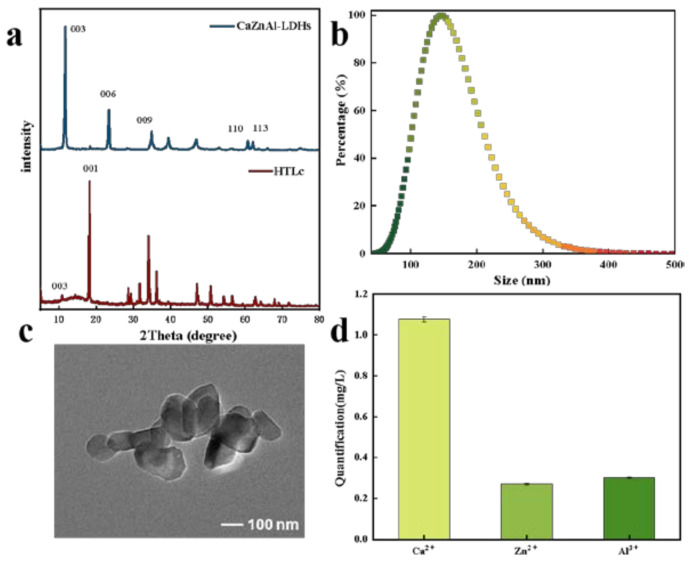
Characterisation of CaZnAl-CO_3_-LDHs: (**a**) XRD patterns of CaZnAl-CO_3_-LDHs and HTLc. (**b**) The particle size distribution of CaZnAl-CO_3_-LDHs platelets. (**c**) TEM images of CaZnAl-CO_3_-LDHs. (**d**) The content of Ca^2+^, Zn^2+^, Al^3+^ in the CaZnAl-CO_3_-LDHs.

Figure 1a shows the XRD patterns of CaZnAl-CO_3_-LDHs and the products after calcination of CaZnAl-CO_3_-LDHs at 280 °C for 5 min by simulating a melt press procedure. As shown in Figure 1a, a series of [003], [006], [009], [110] and [113] shot peaks are narrow, sharp and symmetrical, indicating that the CaZnAl-CO_3_-LDHs are well-crystallized and have the standard XRD patterns of hydrotalcite structures (JCPDS 41-0219). The highest diffraction peak appears at 2*θ* = 11.67° and d_(003)_ = 0.758 nm, showing that the CaZnAl-CO_3_-LDHs, have a typical hexagonal crystal structure. A new diffraction peak appears at 2*θ* = 18.31° after calcination at 280 °C, and the intensity of this peak increases with the increasing temperature (Figure S1). This diffraction peak could be belonged to (001) crystallographic diffraction peak of oxide. Based on the XRD pattern, it can currently be assumed that the material is made up of two composite phases (HTLc, I) of hydrotalcite and its oxide (II), with crystalline spacings of 0.758 nm for phase I and 0.484 nm for phase II. The shrinking of the laminate induced by the loss of interlayer water and the dehydroxylation of the laminate causes phase I to progressively transition into phase II [21]. Transmission electron microscopy (TEM) images (Figure 1c) show a primary plate diameter in the range of 100~200 nm, which is consistent with the average particle size of ~140 nm obtained from dynamic light scattering (DLS) analysis (Figure 1b). ICP was used to determine the concentration of each metal element in the CaZnAl-CO_3_-LDHs, and the molar ratio of Ca/Zn/Al in the synthesized hydrotalcite was 2.7:0.12:1.3, as shown in Figure 1d.

**Figure 2:**
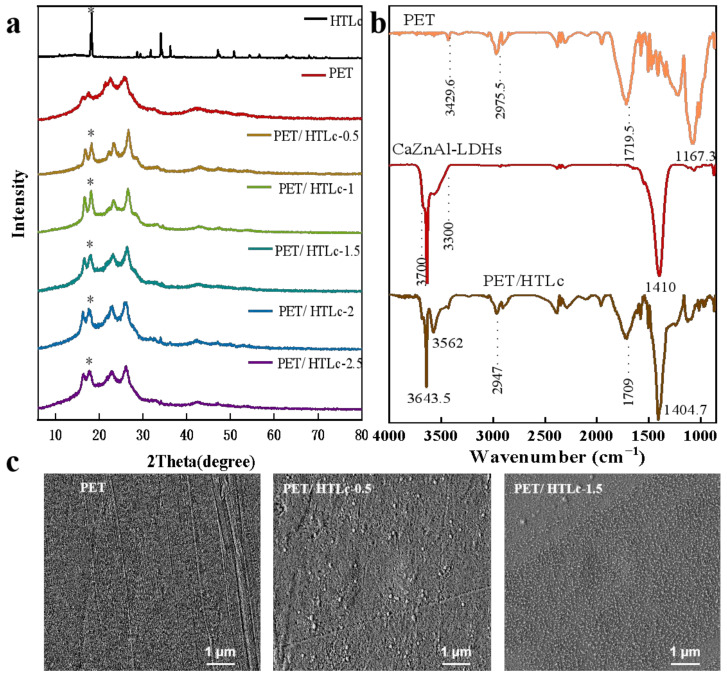
Morphology and structure of PET/HTLc films: (**a**) XRD patterns of a series of PET/HTLc composite films, asterisks denote the 001 reflections. (**b**) FT-IR spectra of PET, CaZnAl-LDHs and PET/HTLc film. (**c**) SEM images of PET, PET/HTLc-0.5 and PET/HTLc-1.5.

Figure 2a shows the XRD patterns of the composite films formed by combining different ratios of LDHs with PET. For pure PET film, there is no obvious diffraction peak near 2*θ* = 18.31°, while the diffraction peak near 2*θ* = 18.31° for the composite film becomes sharper and clearer as the proportion of HTLc increases, which indicates that LDHs have been successfully bonded to PET during high-temperature hot pressing. It also demonstrates that the precursor LDHs have become a two-phase mixture (HTLc) of hydrotalcite and its oxide in the composite film.

The SEM images (Figure 2c) of PET film and a series of PET/HTLc composite films show that the surface of pure PET film is smooth, and small particles with uniform distribution appear in the composite films. With the increasing content of hydrotalcite, the distribution of hydrotalcite in the composite film is gradually dense, which further illustrates the good combination of hydrotalcite and PET in the composite films.

**Scheme 1:**
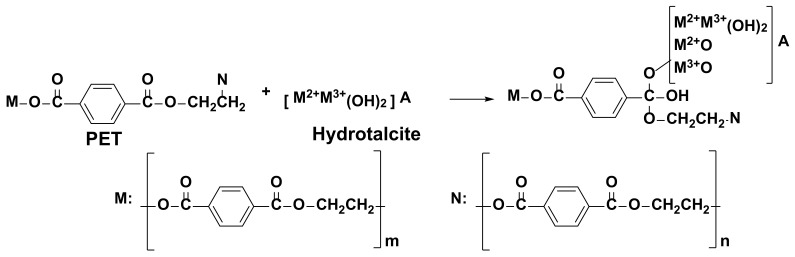
Possible mechanism of hydrotalcite bonding with PET.

The FT-IR spectra of pure PET, CaZnAl-LDHs and PET/HTLc film can be observed in Figure 2b. In PET, the absorption peak at 3429.6 cm^−1^ is the O-H stretching vibration of the terminal group of diethylene glycol. The narrow absorptions of aliphatic C-H stretching vibration and carbonyl C=O stretching occurrence at 2975.5 cm^−1^ and 1719.5 cm^−1^. In addition, the absorption peak at 1167.3 cm^−1^ is the band in the skeletal ring region, indicating aromatic 1, 4-substitution. In CaZnAl-CO_3_-LDHs, the broad and strong band between 3300 and 3700 cm^−1^ is on account of the O-H stretching mode of layer hydroxyl groups and inter-layer water molecules. The narrow absorptions at 1410 cm^−1^ are on account of the *v*_3_ stretching vibration of the interlayer carbonate anions. The IR spectrum of the PET/HTLc film also shows the characteristic absorption peaks of hydroxyl groups of hydrotalcite laminates and interlayer water hydroxyl groups at 3643.5 cm^−1^, the characteristic peaks of O-H stretching vibrations of diethylene glycol terminal groups at 3584.9 cm^−1^, the characteristic peaks of aliphatic C-H stretching vibrations in PET at 2947 cm^−1^ and the characteristic peak of CO_3_^2−^ in CaZnAl-CO_3_-LDHs at 1404.7 cm^−1^. However, unlike pure PET and CaZnAl-CO_3_-LDHs, the metal hydroxide absorption peak appears at 3562 cm^−1^, and the migration of the carbonyl band to a lower wave number to 1709 cm^−1^, which indicates the breakage of the carbonyl bond. The inferred reason is shown in Scheme 1. Under high temperature and pressure, the metal group in hydrotalcite interacts with the carbonyl group C=O on the ester in PET, resulting in the breakage of the C=O bond in PET and the formation of new hydroxyl and metal-oxygen bonds, which also explains more clearly the binding mechanism of hydrotalcite to PET [22]. The XPS pattern (Figure S2) shows that the data from PET/HTLc film could be fitted with four peaks for O1s, indicating the formation of metal–oxygen bonds, which is consistent with the IR results in Figure 2b. Table 1 shows the film thickness obtained from measurements at three randomly selected locations. The TGA curve (Figure S3a) showed that the thermal stability of the PET/HTLc composite film was enhanced compared with the pure PET film at high temperatures. Since HTLc was fully exfoliated and dispersed in the polymer matrix, the interfacial interaction with PET molecular chains was enhanced; thus, it played a significant role in hindering and inhibiting the thermal degradation of PET molecules. Figure S3b show typical DSC thermograms obtained for PET and PET/HTLc samples. Glass transition temperatures (T_g_), cold crystallization temperature (T_cc_), melting points (T_m_), cold crystallization enthalpy (Δ*H*_cc_), melting enthalpy (Δ*H*_m_) and degree of crystallinity (*χ*_c_) heat of cold crystallization are shown in Table S1, for each material. It can be seen that with the addition of HTLc, the T_g_, T_cc_, T_m_ and *χ*_c_ of the samples were increased slightly. After the addition of the inorganic modified powder, the energy required for crystallization from the glassy state is higher, and the energy required for the chain segments to enter the crystalline phase is elevated, leading to more difficult crystallization. This may be due to the fact that HTLc plays an obvious role in heterogeneous nucleation, which increases the crystallization temperature of PET.

### 3.2. Gas Barrier Performance

**Figure 3:**
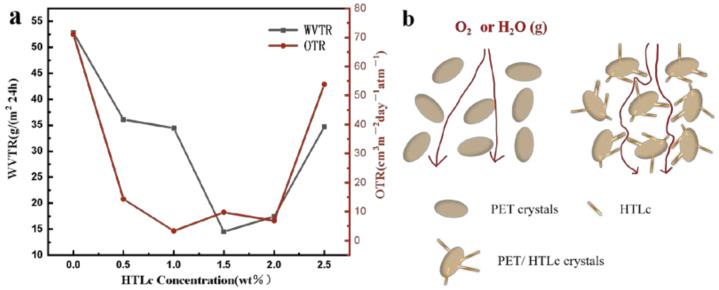
Barrier to oxygen and water vapor. (**a**) Water vapor transmission rate (WVTR) and oxygen transmission rate (OTR) curves for a series of PET/HTLc films (**b**) Illustration of the gas barrier film of the PET/HTLc nanocomposites.

The oxygen transmission rate (OTR) and water vapor transmission rate (WVTR) of pure PET were 71.08 cm^3^m^−2^day^−1^atm^−1^ and 52.86 g/(m^2^ 24 h), respectively, as shown in Figure 3a. With the increase in HTLc content, both OTR and WVTR started to decrease. The lowest OTR of PET/HTLc-1 was 3.36 cm^3^m^−2^day^−1^atm^−1^, which decreased by 95.27% compared with pure PET. The lowest WVTR of PET/HTLc-1.5 was 14.49 g/(m^2^ 24 h), which decreased by 72.58% compared with pure PET. The decrease in film permeability may be related to three reasons: (1) the bending effect caused by the addition of nanoplates, (2) changes in the properties of the polymer matrix (e.g., free volume of the polymer, crystallinity and nucleation effect of the particles), and (3) modification of the adsorption sites of the permeable molecules [23,24]. The interfacial interactions between the building blocks are crucial for the preparation and performance of the hybrid films. The gas barrier properties of PET/HTLc films changed in this work, which was mostly attributable to the bending effect created by the addition of HTLc nanoflakes resulting in the tree-fork effect generated in the composite films. As shown in Scheme 1, the -C-O- bonds in PET/HTLc permit multiple HTLc forks to “grow” around the PET crystal (Figure 3b). When O_2_ and H_2_O (g) diffuse in this structure, it is easily obstructed by these forks, extending the diffusion path of gas molecules [25]. However, when the content of HTLc continued to increase, both OTR and WATR started to increase, although they were still lower than the pure PET transmission, which could be due to the aggregation of HTLc, and some defects appeared on the interface of the composite films [26]. The above suggests that HTLc nanosheets play an important role in the enhancement of barrier properties by extending the transport path of gas molecules in the hybrid film [27,28].

### 3.3. UV-Blocking Performance and the Light Aging Test

**Figure 4:**
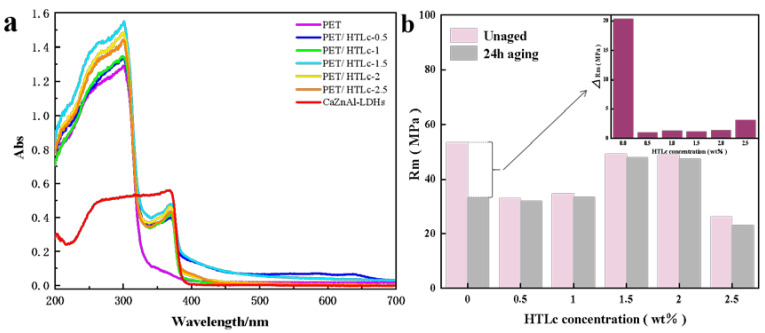
UV light resistance of PET/HTLc films: (**a**) UV absorption curves of PET/HTLc films. (**b**) Tensile strength of PET/HTLc films before and after 24 h UV aging.

Currently, some merchants add UV-blocking material to the packaging film, such as carbon black, which makes the film blackish and unattractive, limiting its application in many fields. In this study, the UV-blocking ability of pure PET and PET composites was characterized by measuring the UV absorbance (Abs) in the wavelength range of 200~800 nm. Figure 4a shows that the main UV absorption range of pure PET is between 200 and 300 nm, and the absorption range of the composite film is expanded to 200 to 380 nm after the addition of various mass fractions of HTLc. Moreover, the UV absorption increases by 20.81% in the 200 nm to 380 nm, indicating that the introduction of HTLc has improved the UV shielding ability of PET films [16,29]. This is due to UV light absorption by ZnO present in the (II) phase described in XRD, as well as the scattering effect of the inorganic powder. Therefore, it can mitigate the negative effects of photolysis after extended UV exposure and effectively minimize food spoiling caused by light exposure.

In order to evaluate the photo-stability quantitatively, the change in tensile strength of PET/HTLc films was determined after exposure to artificially accelerated photoaging simulated UV irradiation for 24 h. It can be seen from the Figure 4b that although the tensile strength of the pure PET film was up to 53.395 MPa before UV aging, it rapidly decreased to 33.021 MPa after irradiation, and the ΔRm was 20.374 MPa, indicating the poor UV stability of pure PET. The effect of UV radiation on the polymer is accomplished by the free radicals generated by molecular bond cleavage. Excessive UV irradiation causes damage to the fiber-forming polymer molecules, leading to a decrease in their mechanical properties [30]. Furthermore, the ΔRm of a series of composite films is significantly smaller than that of pure PET films, and the ΔRm of PET/HTLc-0.5 is the smallest at 1.046, indicating that the presence of HTLc can significantly retard the photoaging process of PET. It is worth noting that the addition of HTLc causes a slight decrease in the tensile strength of the laminated film compared to pure PET, which may be due to the dispersion of HTLc in PET under high-temperature pressure conditions causing a lower degree of damage to the PET films. Nevertheless, the composite films still satisfy the mechanical strength standards for food packaging film when compared to the ASTM D882 and GB/T 28117 standards for tensile strength.

### 3.4. Antibacterial Performance

**Figure 5:**
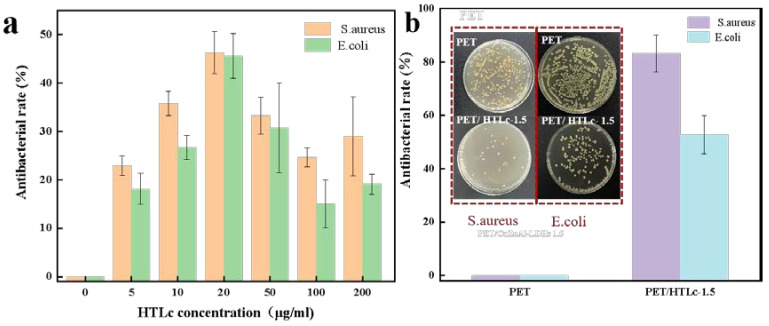
Antibacterial performance of HTLc and PET/HTLc-1.5: (**a**) Antibacterial rates of HTLc against *S. aureus* and *E. coli* at different concentrations. (**b**) Antibacterial rates of PET/HTLc-1.5 film against *S. aureus* and *E. coli*.

In order to verify the antibacterial performance of the composite films, the antibacterial efficacy of different HTLc precursors was first evaluated with Escherichia coli (*E. coli*, Gram-negative) and Staphylococcus aureus (*S. aureus*, Gram-positive). The results are shown in Figure 5a. It can be seen that as the concentration of HTLc increases, the inhibition rate of both bacteria increases and then decreases, and there is an optimal inhibition concentration. The maximal inhibition rate of HTLc for *S. aureus* and *E. coli* is 46.26% and 45.63%, respectively, at 20 g/mL (ω = 0.02%).

Furthermore, the antibacterial effect of the composite film PET/HTLc-1.5 was evaluated, which had the best gas barrier performance and UV light resistance in the previous tests, and the results are shown in Figure 5b. By using the pure PET film as the negative control, the inhibition rate of PET/HTLc-1.5 was 83.19% for *S. aureus* and 52.75% for *E. coli*, indicating that the presence of HTLc enhanced the inhibition ability of the composite film. 

**Figure 6:**
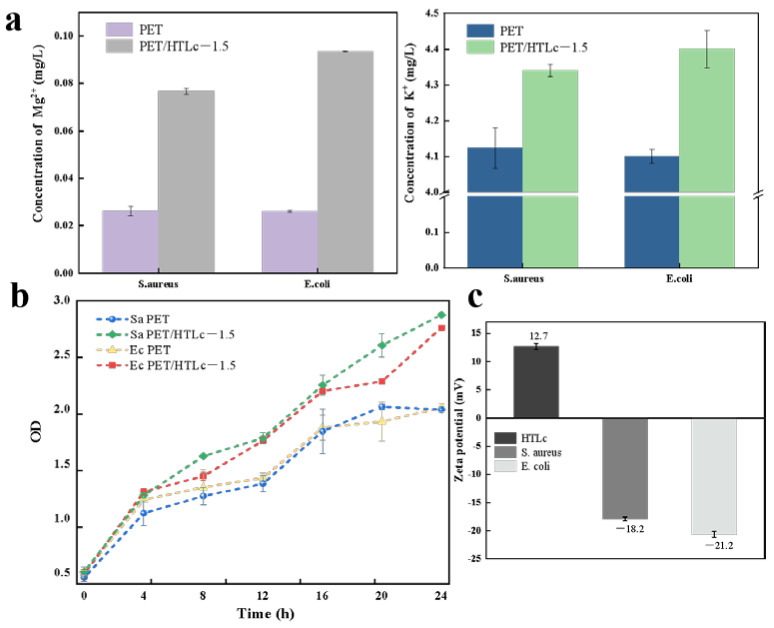
Bacterial inhibition mechanism of PET/HTLc-1.5 film: (**a**) Effect of PET/HTLc-1.5 on the extracellular Mg^2+^, K^+^ content of *S.aureus* and *E.coli*. (**b**) Effect of PET/HTLc-1.5 on the extracellular protein content of *S. aureus* and *E. coli*. (**c**) *z*-potentials of HTLc, *S. aureus* and *E. coli*.

In order to further investigate this inhibition principle, the extracellular metal ion content and protein content of bacteria were examined after the action of PET/HTLc-1.5 on *S. aureus* and *E. coli*, respectively [31]. It was found from Figure 6a that the extracellular K^+^ and Mg^2+^ content of the bacteria after 15 h of treatment with PET/HTLc-1.5 are clearly higher than that of the control group. The HTLc in the composite membrane may have altered the permeability of *S. aureus* and *E. coli* cell membranes, leading to the leakage of intracellular ions from the bacteria [32]. By measuring the UV absorbance of the extracellular solution at 562 nm and using the BCA protein concentration assay to calculate its content, greater OD values signify a higher protein concentration. As demonstrated in Figure 6b, the extracellular protein content in the PET/HTLc-1.5 treated group increased significantly as the bacteria incubation time increased, while the PET-treated group experienced less of a change in extracellular protein content. After 24 h, the experimental group’s extracellular protein concentration was noticeably higher than that of the control group. It was again verified that the HTLc in the composite membrane caused serious damage to both bacteria’s cell membranes, resulting in extracellular protein spillage. Gao’s [33,34] research showed that hydrotalcite nanocomposites bind to bacteria through electrostatic interactions, leading to cell membrane damage and rupture, leading to apoptosis. According to the zeta potential (Figure 6c), the z-potential of the HTLc nanocompression site is 12.7 mV, indicating that its surface is positively charged. The bacteria exhibited a negative z-potential (*S. aureus*: −18.2 mV and *E. coli*: −21.2 mV). The large difference in z-potential between HTLc and bacteria results in strong binding of HTLc to the bacterial surface, making PET/HTLc-1.5 easier to interact with bacteria more efficiently.

### 3.5. Migration Testing

**Figure 7:**
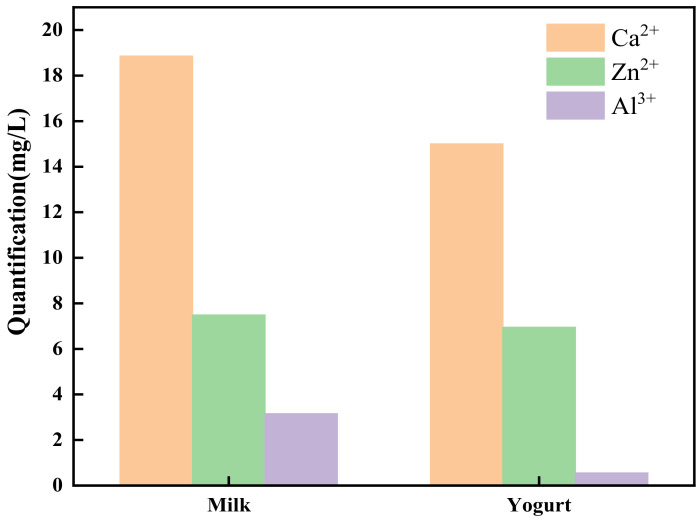
Migration of Ca^2+^, Zn^2+^ and Al^3+^ in PET/HTLc-1.5 film after 10 days in milk and yogurt.

Specific migration limit (SML) is the maximum allowable amount of an additive or class of additives that can migrate from food contact materials and products to the food or food simulant with which they come into contact. It can be expressed as a milligram per kilogram (mg/kg) of migrated material from food or food simulant or milligram per square decimetre (mg/dm^2^) of migrated material from food contact materials and products in contact with food or food simulant [35]. The measurement results of the migration experiment for HTLc are shown in Figure 7. Compared with the dairy samples before the migration experiment, after 10 days, the total amount of Ca migrated in milk and yogurt was 18.8545 and 15.0 mg/kg, and the total amount of Al migrated was 3.1535 and 0.55 mg/kg, respectively. The total amount of migration of Zn, a specially limited metal element mentioned in the regulation, was 7.49 and 6.95 mg/kg, respectively, far less than the SML(Zn): 25 mg/kg [36]. Therefore, it can be said that the PET/HTLc-1.5 composite film meets the standards for the use of additives in food contact materials and products and can be used in the field of food packaging.

## 4. Conclusions

PET/HTLc composite films were prepared by a hot pressing method, and the effect of HTLc content on their structures and properties was investigated. The results of TGA and DSC showed that the addition of HTLc played a significant role in heterogeneous nucleation and improved the thermal stability and crystallinity of PET/HTLc films. Subsequently, The OTR of PET/HTLc-1 was reduced by 95.27%, and the WATR of PET/HTLc-1.5 was reduced by 72.58% compared with pure film. This is due to the unique structure of the PET/HTLc film, where the “tree-fork effect” between the HTLc and the PET extends the path of gas molecules, resulting in a first-class gas barrier performance. In addition, The composite film has enhanced UV barrier qualities due to the addition of inorganic particles HTLc, and the change in tensile strength ΔRm after UV irradiation is minimal. More importantly, PET/HTLc-1.5 possesses certain antibacterial properties, with 83.19% inhibition against *S. aureus* and 52.75% inhibition against *E. coli.* Finally, in the process of simulating the migration of the composite film in dairy products, the migration of Ca, Zn and Al meet the standard, indicating that the composite films can be safely applied to dairy packaging. This work has the potential to be a promising approach to improving PET functional materials.

## Figures and Tables

**Table 1:** Thickness of composite films.

Thickness/μm	PET	PET/HTLc-0.5	PET/HTLc-1	PET/HTLc-1.5	PET/HTLc-2	PET/HTLc-2.5
Area I	182 ± 1.163	185 ± 1.332	185 ± 1.041	182 ± 1.145	187 ± 1.045	191 ± 1.302
Area II	190 ± 1.511	188 ± 1.808	187 ± 1.214	179 ± 2.257	188 ± 1.045	187 ± 1.667
Area III	188 ± 2.055	181 ± 2.012	191 ± 1.507	187 ± 1.076	193 ± 1.056	186 ± 1.205

## Data Availability

The data presented in this study are available on request from the corresponding author.

## Supplementary Materials

The following supporting information can be downloaded at: https://www.mdpi.com/article/10.3390/ma16051857/s1: Figure S1. XRD patterns of CaZnAl-CO3-LDHs at different temperatures; Figure S2. XPS survey spectra of high-resolution scans of O 1s of PET and PET/HTLc; Figure S3. TGA and DSC curve of PET and PET/HTLc; Table S1. DSC results of PET and PET/HTLc.
