# Supplementary material for: A Hydrotalcite-Based PET Composites with Enhanced Properties for Liquid Milk Packaging Applications
Source: Materials (Basel). 2023 Feb 24;16(5):1857. doi: 10.3390/ma16051857 (PMC10004223; doi:10.3390/ma16051857)
Supplement: Supplementary file 1 [file materials-16-01857-s001.zip › materials-2119337-supplementary.pdf]

A hydrotalcite-based PET composites with enhanced properties for liquid milk packaging applications

Xiangnan Feng[1], Xiaomeng Hu[1], Jie Yu[1], Min Zhao[1], Fan Yang[1], Xinrui Wang[1,*], Caili Zhang[1], Yunxuan Weng[1], Jingbin Han[2]

[1] College of Chemistry and Materials Engineering, China National Light Industry, Beijing Technology and Business University, Beijing, 100048, China

[2] State Key Laboratory of Chemical Resource Engineering, Beijing University of Chemical Technology, Beijing, 100029, China

*Correspondence: wangxinrui@th.btbu.edu.cn; Tel: +86 010 68984929

Sample Characterization

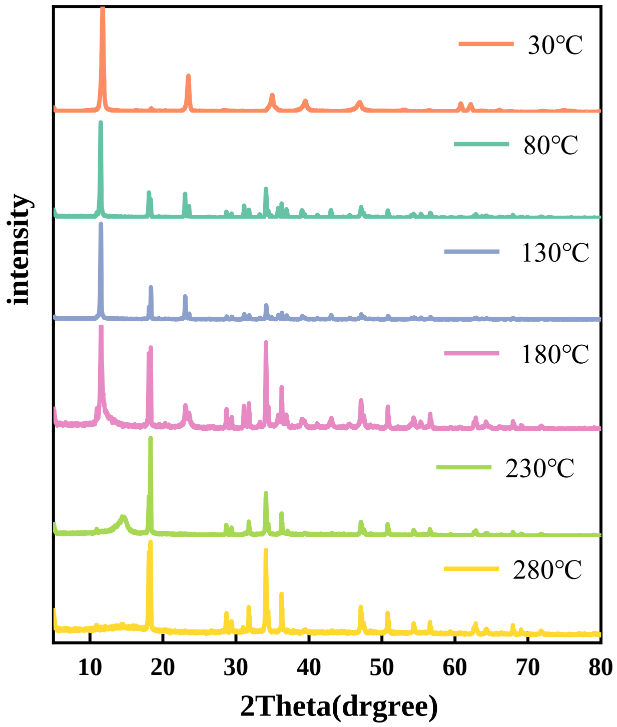

Figure S1. XRD patterns of CaZnAl-CO_3-LDHs at different temperatures

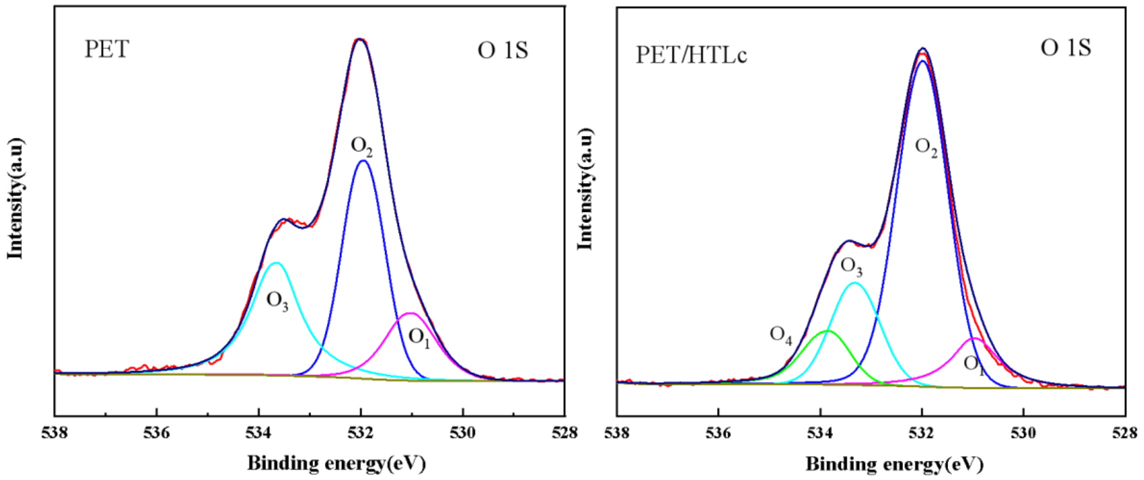

Figure S2 XPS survey spectra of high-resolution scans of O 1s of PET and PET/HTLc.

The O1s spectrum (Figure S2) from the initial PET film showed three main peaks BE at 531.05, 531.95,and 533.7 eV. The data from PET/HTLc film could be fitted with 4 peaks for O1s (531.05, 531.95, 533.3 eV and 533.95), indicating the formation of metal-oxygen bond which is consistent with the IR results in Figure 2b.

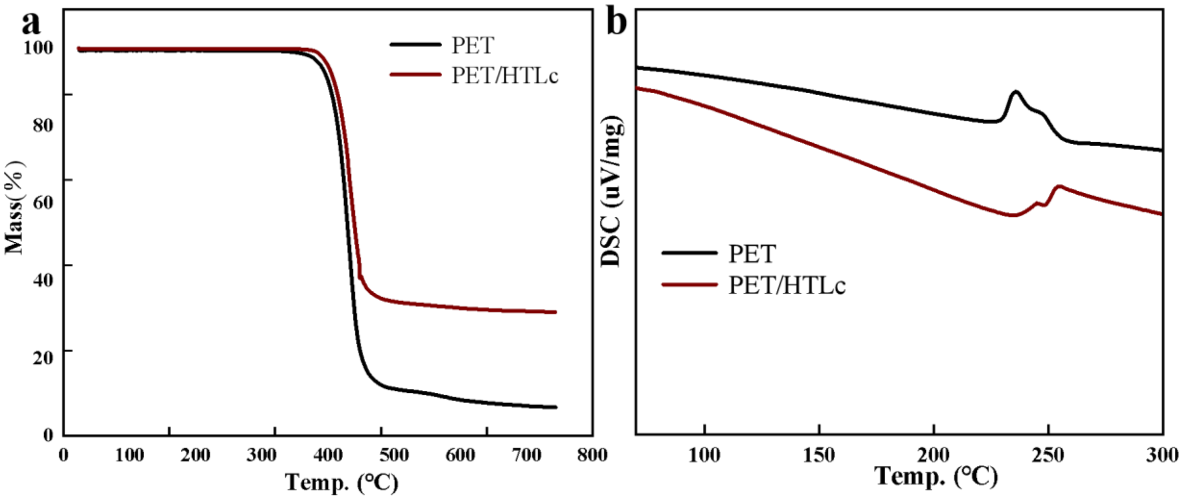

Figure S3 a) TGA and b) DSC curves of PET and PET/HTLc.

The TGA curve (Figure S3a) showed that the thermal stability of the PET/HTLc composite film was enhanced compared with the pure PET film at high temperatures. Since HTLc was fully exfoliated and dispersed in the polymer matrix, the interfacial interaction with PET molecular chains was enhanced, thus it played a significant role in hindering and inhibiting the thermal degradation of PET molecules. Figure S3b show typical DSC thermograms obtained for PET and PET/HTLc samples. Glass transition temperatures (T_g), cold crystallization temperature (T_{cc}), melting points (T_m), cold crystallization enthalpy (ΔH_{cc}), melting enthalpy (ΔH_m) and degree of crystallinity (χ_c) heat of cold crystallization are shown in Table S1, for each material. It can be seen that with the addition of HTLc, T_g, T_{cc}, T_m and χ_c

of the samples were increased slightly. After the addition of the inorganic modified powder, the energy required for crystallization from the glassy state is higher and the energy required for the chain segments to enter the crystalline phase is elevated, leading to more difficult crystallization. This may be due to the fact that HTLc plays an obvious role in heterogeneous nucleation, which increases the crystallization temperature of PET.

Table S1 DSC results of PET and PET/HTLc

Sample	T_g/°C	T_{cc}/°C	T_m/°C	ΔH_{cc} (J/g)	ΔH_m (J/g)	χ_c (%)
PET	73.88	225.88	237.78	21.28	23.74	1.76
PET/HTLc	78.99	234.56	254.97	25.03	31.86	4.88
